# Serum Uric Acid Levels and Renal Function in Untreated Hypertensive Patients

**DOI:** 10.7759/cureus.100793

**Published:** 2026-01-05

**Authors:** Georgios Vlachopanos, Panagiotis Theofilis, Katerina Vordoni, Despina Smirloglou, Despina Karasavvidou, Kosmas Pappas, Vasilis Tsimihodimos, Rigas Kalaitzidis

**Affiliations:** 1 Center for Nephrology "G. Papadakis", General Hospital of Nikaia-Piraeus "Agios Panteleimon", Piraeus, GRC; 2 Department of Nephrology "C. Katsinas", General Hospital of Ptolemaida "Mpodosakeio", Ptolemaida, GRC; 3 Department of Internal Medicine, Faculty of Medicine, University of Ioannina, Ioannina, GRC

**Keywords:** chronic kidney disease (ckd), estimated glomerular filtration rate (egfr), renal function, serum uric acid (sua), systemic arterial hypertension

## Abstract

Background and aim

Serum uric acid is considered to be a risk factor contributing to the progression of cardiovascular disease. In this cross-sectional study that used existing data, we evaluated possible correlations between serum uric acid levels and renal function and the hypothesis that elevated serum uric acid levels may be associated with worse renal function in untreated patients with arterial hypertension.

Patients and methods

The study involved 446 hypertensive patients (226 men, 220 women) of 52.7 ± 12.6 years of age who visited our Outpatient Hypertension Clinic and did not receive any antihypertensive therapy.

Results

Patient separation into three groups according to serum uric acid (SUA) levels (SUA: <6, 6-8, >8 mg/dl) showed that patients with lower serum uric acid levels had lower creatinine levels and higher estimated glomerular filtration rate (eGFR), as calculated by the Chronic Kidney Disease Epidemiology Collaboration (CKD-EPI) equation, and vice versa. A statistically significant correlation was observed between serum uric acid levels and either serum creatinine levels (r=0.54, p<0.001) or eGFR (r=-0.19, p<0.01).

Conclusions

Untreated hypertensive subjects with higher serum uric acid levels exhibit reduced renal function compared with hypertensive individuals with lower serum uric acid levels.

## Introduction

High serum uric acid (SUA) levels are common in the general population and are correlated with a plethora of comorbidities such as cardiovascular disease (e.g., coronary artery disease), metabolic disturbances (e.g., insulin resistance), and chronic kidney disease (CKD), irrespective of the coexistence of gout or not [1]. Although it has been questioned whether this correlation reflects true causation or is just a spurious epiphenomenon due to residual confounding, data from studies assessing the biologic actions of SUA strongly support the former hypothesis [2]. SUA is a well-known extracellular antioxidant; however, it becomes a potent pro-oxidant factor when found in an inflammatory milieu like the one that exists in patients with the metabolic syndrome. Hyperuricemia is common in patients with arterial hypertension (it reaches a prevalence of 22%-25% in uncontrolled hypertension) and is an established risk factor for the development of arterial hypertension [3]; in a systematic review and meta-analysis of 18 prospective cohort studies with 55,607 participants included, it was found that for every 1 mg/dl increase in SUA levels, the pooled risk ratio for incident hypertension increased by 13% after adjustment for potential confounding factors (RR: 1.13, 95% CI: 1.06-1.20) [4].

Experimental studies have reported that high SUA levels may have a rather early impact on arterial hypertension [5-6]. That leads over time to the accumulation of microvascular lesions which, ultimately, mediate hypertension irrespective of SUA [7]. As a consequence, adolescent age has been selected as the research focus regarding the relationship between SUA levels and arterial hypertension, because endothelial dysfunction and inflammatory lesions have not been developed yet at such an early age. In a study of 125 patients aged six to 18 years who were evaluated for new-onset, untreated hypertension, it was reported that SUA >5.5 mg/dl was found in 89% of primary hypertension patients [8]. Furthermore, SUA levels were directly correlated with systolic (r=0.80, p=0.0002) and diastolic (r=0.66, p=0.0006) blood pressure (BP) in controls and in subjects with primary hypertension and that effect was independent of renal function.

Studies on treatment-naïve hypertensive patients have demonstrated that elevated SUA levels are significantly higher in untreated hypertensives and that are significantly associated with components of the metabolic syndrome such as waist circumference, low-density lipoprotein (LDL), high-density lipoprotein (HDL), and triglycerides [9-11]. However, data on the relationship between SUA and renal function in untreated patients with arterial hypertension are scarce. We hypothesized that hyperuricemia may be associated with decreased renal function in untreated hypertensive patients and we performed a cross-sectional study to evaluate that hypothesis. The assessment of the relationship between SUA and creatinine and between SUA and estimated glomerular filtration rate (eGFR) after patient separation into three groups according to SUA levels (<6, 6-8, >8 mg/dl) was set as primary outcome of the study. Secondary outcome of the study was to clarify the correlation between SUA and creatinine or eGFR. This article was previously presented as a meeting abstract at the 28th European Meeting on Hypertension and Cardiovascular Protection on June 10, 2018.

## Materials and methods

Patients

The present study was a cross-sectional study that used already existing data. These data were collected from January 1, 2018, until April 30, 2018, and concerned 446 consecutive patients who attended the outpatient clinic of the Department of Internal Medicine, Faculty of Medicine, University of Ioannina, Greece. The inclusion criteria were the following: (a) age ≥ 18 years old; (b) patients with recently diagnosed primary hypertension; (c) patients who did not receive any antihypertensive treatment and (d) sufficient knowledge of the Greek language. Active gout or a history of gout was an exclusion criterion. Hypertension was defined as office systolic blood pressure (SBP) values ≥140 mmHg and/or diastolic blood pressure (DBP) values ≥90 mmHg as per the European Society of Hypertension guidelines [12]. BP was classified as normal (SBP: 120-129 mmHg, DBP: 80-84 mmHg), high-normal (SBP: 130-139 mmHg, DBP: 85-89 mmHg), grade 1 hypertension (SBP: 140-159 mmHg, DBP: 90-99 mmHg), grade 2 hypertension (SBP: 160-179 mmHg, DBP: 100-109 mmHg), or grade 3 hypertension (SBP: ≥180 mmHg, DBP: ≥ 110 mmHg) according to office BP. Confirmation of hypertension required three BP measurements with one minute interval between them. We retrieved and recorded all available demographic, clinical and laboratory features through electronic health files. Primary outcome of the study was to assess the relationship between SUA and creatinine and between SUA and estimated glomerular filtration rate (eGFR) after patient separation into three groups according to SUA levels (<6, 6-8, >8 mg/dl). Secondary outcome of the study was to draw the correlation between SUA and creatinine or eGFR (and visualize it by a line diagram). Enzymatic assay methods in automated analyzers were used for creatinine and SUA estimation (creatininase-peroxidase and uricase-peroxidase, respectively). The study was performed in accordance with the ethical principles of the Declaration of Helsinki.

Statistical analysis

Demographic, clinical and laboratory data are presented as mean value ± standard deviation or as percentage %. For continuous variables, analysis of variance (ANOVA) was used for overall statistical significance assessment of the three stratified groups and t-test was used for between-group comparisons. The correlations between serum uric acid levels and either serum creatinine levels or eGFR (as calculated by Chronic Kidney Disease Epidemiology Collaboration (CKD-EPI) equation) were done using Pearson’s r correlation coefficient [13]. The level of statistical significance was set at p<0.05. Statistical analysis was performed with IBM SPSS Statistics for Windows (version 22.0, Armonk, NY, IBM Corp.).

## Results

Baseline characteristics of the study population are shown in Table 1. Mean age of the population was 52.7±12.6 years, and 51% of them were males (226 males/220 females). Mean body mass index (BMI) was 26.90±4.30 kg/m^2^, and mean waist circumference was 97.30±11.10 cm. Mean SBP was 149.05±22.70 mmHg, whereas mean DBP was 94.70±14.10 mmHg. Mean serum electrolyte concentrations are summarized in Table 2. It is noteworthy that mean SUA levels were 4.90±1.48 mg/dl. Biochemical test results are depicted in Table 3. Mean glucose levels were 98±21 mg/dl, and mean urea levels were 32.00±9.10 mg/dl. Mean creatinine levels were 0.92±0.17 mg/dl with the corresponding mean eGFR levels being 85.50±13.70 ml/min/1.73 m^2^. Mean total cholesterol levels were 218±53 mg/dl, mean triglyceride levels were 134±72 mg/dl, mean HDL cholesterol levels were 48±12 mg/dl, and mean LDL cholesterol levels were 143±48 mg/dl.

**Table 1:** Demographic and clinical patient characteristics. Data are presented as mean value±standard deviation or as percentage %. BMI, body mass index; DBP, diastolic blood pressure; SBP, systolic blood pressure.

Parameter	Total (n=446)
Age (years)	52.7±12.6
Gender (% male)	226 (51%)
BMI (kg/m^2^)	26.09±4.30
Waist circumference (cm)	97.30±11.10
SBP (mmHg)	149.50± 22.70
DBP (mmHg)	94.70±14.10
Heart rate/min	73.50±12.30

**Table 2:** Serum electrolyte and uric acid concentrations in study population. Data are presented as mean value±standard deviation. Na^+^, sodium; K^+^, potassium; Mg^+2^, magnesium; Ca^+2^, calcium; Cl^-^, chloride; PO4^-3^, phosphate.

Parameter	Total (n=446)	Reference range
Na^+^ (mEq/L)	141.80±1.90	135.0-145.0
K^+^ (mEq/L)	4.50±0.34	3.5-5.2
Mg^+2^ (mg/dl)	1.69±0.16	1.7-2.2
Ca^+2^ (mg/dl)	9.48±0.40	8.5-10.2
Cl^-^ (mg/dl)	104.70±2.40	96.0-106.0
PO4^-3^ (mg/dl)	3.11±0.46	2.5-4.5
Uric acid (mg/dl)	4.90±1.48	3.0-6.0

**Table 3:** Metabolic profile in study population. Data are presented as mean value±standard deviation. eGFR, estimated glomerular filtration rate; HDL, high-density lipoprotein; HOMA-IR, Homeostatic Model Assessment for Insulin Resistance; LDL, low-density lipoprotein.

Parameter	Total (n=446)	Reference range
Glucose (mg/dl)	98±21	70-100
Insulin (Mu/ml)	9.80±5.60	2.0-25.0
HOMA-IR index	2.40±1.80	0.5-2.5
Urea (mg/dl)	32.00±9.10	15.0-45.0
Creatinine (mg/dl)	0.92 ±0.17	0.7-1.3
eGFR (ml/min/1.73m^2^)	85.50±13.70	90.0-120.0
Total cholesterol (mg/dl)	218±53	<200
Triglycerides (mg/dl)	134±72	<150
HDL cholesterol (mg/dl)	48±12	40-80
LDL cholesterol (mg/dl)	143±48	<100

The stratification of patients into three groups according to SUA levels (<6, 6-8, >8 mg/dl) showed that patients with SUA levels had lower creatinine levels and higher eGFR, and vice versa, and this result achieved statistical significance (Table 4). Patients with SUA levels <6 mg/dl had a mean creatinine of 0.88±0.13 mg/dl and a mean eGFR of 87.60±12.40 ml/min/1.73 m^2^, patients with SUA levels between 6 and 8 mg/dl had a mean creatinine of 1.03±0.18 mg/dl and a mean eGFR of 81.20±13.90 ml/min/1.73m^2^, whereas patients with SUA levels >8 mg/dl had a mean creatinine of 1.10±0.20 mg/dl and a mean eGFR of 79.00±19.90 ml/min/1.73 m^2^.

**Table 4:** Uric acid, creatinine and eGFR levels in three groups stratified according to uric acid levels (UA <6, 6-8, >8mg/dl). Data are presented as mean value±standard deviation. ANOVA test was used for assessing overall statistical significance of the three stratified groups, and t-test was used for between-group comparisons (superscripts a-i) as follows: a) Uric acid level comparison between Group 1 and Group 2 (p=0.001), b) Uric acid level comparison between Group 1 and Group 3 (p=0.001), c) Uric acid level comparison between Group 2 and Group 3 (p=0.001), d) Creatinine level comparison between Group 1 and Group 2 (p=0.001), e) Creatinine level comparison between Group 1 and Group 3 (p=0.001), f) Creatinine level comparison between Group 2 and Group 3 (p=0.007), g) eGFR level comparison between Group 1 and Group 2 (p=0.001), h) eGFR level comparison between Group 1 and Group 3 (p=0.001), i) eGFR level comparison between Group 2 and Group 3 (p=0.55). Value of statistical significance test was set at p<0.05.  ANOVA, analysis of variance; eGFR, estimated glomerular filtration rate; t-test, Student's two sample t-test.

Parameter	Group 1 (n=210): Uric acid< 6	Group 2 (n=154): 6≥Uric acid≤8	Group 3 (n=82): Uric acid>8	ANOVA test p value
Uric acid (mg/dl)	4.25±1.00^a,b^	6.60±0.50^a,c^	8.50±0.34^b,c^	0.001
Creatinine (mg/dl)	0.88±0.13^d,e^	1.03±0.18^d,f^	1.10±0.20^e,f^	0.001
eGFR (ml/min/1.73m^2^)	87.60±12.40^g,h^	81.20±13.90^g,i^	79.00±19.90^h,i^	0.01

**Figure 1:**
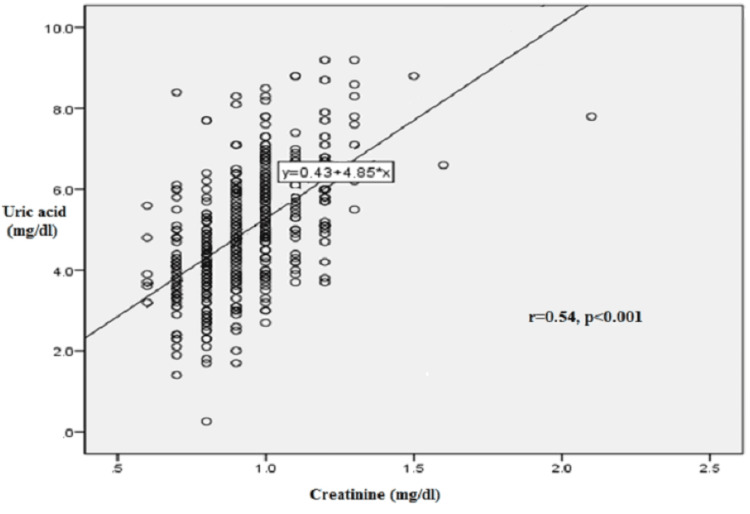
Line diagram of the correlation between serum uric acid and creatinine. Correlation coefficient r was 0.54 (p<0.001) denoting statistically significant agreement. r, Pearson's correlation coefficient.

**Figure 2:**
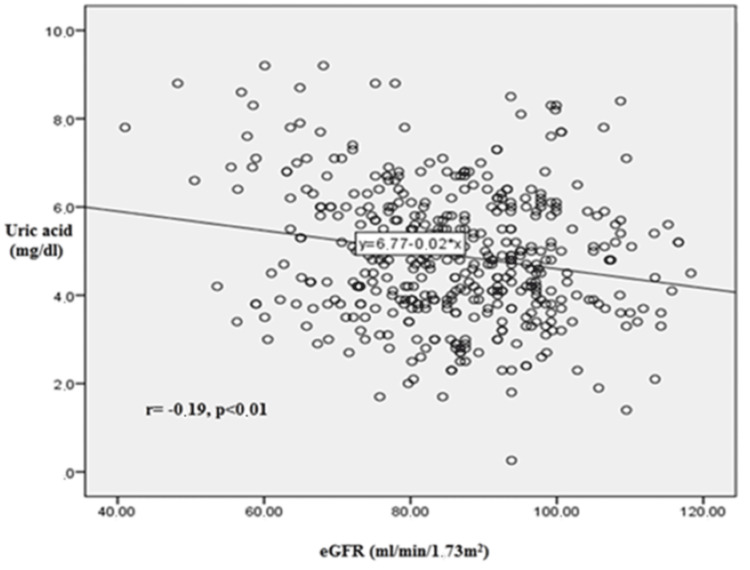
Line diagram of the correlation between serum uric acid and eGFR (as calculated by the CKD-EPI equation). Correlation coefficient r was -0.19 (p<0.01) denoting statistically significant agreement. eGFR, estimated glomerular filtration rate; r, Pearson's correlation coefficient; CKD-EPI: Chronic Kidney Disease Epidemiology Collaboration.

A statistically significant positive correlation was found between SUA and serum creatinine levels, as shown in Figure 1. The Pearson correlation coefficient was r= 0.54 (p<0.001), indicating a moderate-to-strong positive relationship, with higher SUA levels associated with higher serum creatinine values. Conversely, a significant negative correlation was observed between serum uric acid levels and eGFR, as illustrated in Figure 2. The Pearson correlation coefficient was r=-0.19 (p<0.01), suggesting a weak inverse association, whereby increasing SUA concentrations corresponded with a decline in renal function as measured by eGFR. These findings support the hypothesis that elevated uric acid levels are associated with impaired renal function in untreated hypertensive patients.

## Discussion

The present study aimed to investigate the relationship between SUA levels and renal function in a population of untreated hypertensive patients. Our findings suggest a statistically significant association between elevated SUA levels and reduced renal function, as indicated by higher serum creatinine levels and lower eGFR. These results are consistent with previous studies highlighting the potential role of hyperuricemia as a contributing factor in the development and progression of CKD, particularly in patients with coexisting hypertension.

The role of uric acid in renal physiology and pathophysiology has long been a subject of debate. Although traditionally viewed as a marker rather than a mediator of renal impairment, growing evidence supports its causal involvement in renal damage. Experimental animal models have demonstrated that uric acid induces afferent arteriolopathy, glomerular hypertension, and tubulointerstitial fibrosis through mechanisms involving endothelial dysfunction, oxidative stress, and activation of the renin-angiotensin-aldosterone system (RAAS) [2,5,14]. In our study, the observation that higher SUA levels correlated positively with creatinine and negatively with eGFR further supports the hypothesis that hyperuricemia may have a deleterious impact on renal function, even in the early stages of hypertension.

Several mechanisms may explain the nephrotoxic effects of uric acid. Firstly, uric acid reduces endothelial nitric oxide availability, impairing vasodilation and promoting vascular smooth muscle proliferation [15]. Secondly, it induces pro-inflammatory pathways and oxidative stress in renal tubular cells and endothelial cells [6]. Thirdly, uric acid promotes RAAS activation, which contributes to renal vasoconstriction and increased intraglomerular pressure [7]. These pathophysiological mechanisms could account for the observed renal dysfunction in hypertensive patients with elevated SUA in our cohort. Moreover, histological lesions are described in the kidneys of more than 90% of patients with long-standing gout [16]. These include chronic tubulointerstitial fibrosis and medullary uric acid crystal deposits [17]. In a more recent study, the deposition of uric acid crystals in the renal medulla was found by ultrasound in 36% of patients with gout [18].

Our results are in line with findings from Feig and Johnson, who first demonstrated in adolescents with newly diagnosed hypertension that elevated SUA was not only common but independently associated with increased blood pressure and target organ damage [8]. In a subsequent interventional study, the same group showed that lowering SUA with allopurinol led to significant reductions in both systolic and diastolic blood pressure in adolescents with newly diagnosed hypertension [19]. These findings suggest that hyperuricemia may not merely be a bystander but an active contributor to hypertensive nephropathy.

Epidemiological studies further support the association between SUA and renal function. A large prospective cohort study by Obermayr et al. in over 21,000 healthy individuals found that higher SUA levels predicted the development of new-onset kidney disease over an average follow-up of seven years [20]. Likewise, Weiner et al. reported that higher baseline SUA levels were independently associated with increased risk of kidney function decline in participants of the Atherosclerosis Risk in Communities (ARIC) study [21]. Our data, which show reduced eGFR in hypertensive patients with SUA >6 mg/dl, are consistent with these longitudinal observations.

It is noteworthy that in our population, all patients were treatment-naïve, thus confounding by antihypertensive or urate-lowering medications was eliminated. Certain antihypertensive drugs, particularly diuretics, are known to increase uric acid levels, potentially masking the true nature of the relationship between SUA and renal function [22]. By focusing on untreated individuals, our study design provides clearer insight into the baseline association between SUA and kidney function without pharmaceutical interference.

Another notable finding in our analysis is that the correlation between uric acid and eGFR, while statistically significant, was at best modest in strength. This could suggest that while SUA plays a role in renal impairment, it is likely only one of multiple contributing factors, some of which may be more important for renal disease progression. It is well established that obesity, insulin resistance, dyslipidemia, and systemic inflammation, all components of the metabolic syndrome, contribute to both hyperuricemia and CKD [23]. Indeed, it has been proposed that SUA is a marker for metabolic dysregulation rather than a direct cause of renal disease [24].

Although small, early interventional trials suggested that urate-lowering therapy could be renoprotective in selected populations, larger randomized control trials that followed failed to show that hyperuricemia treatment may slow chronic kidney disease progression. Siu et al. found that lowering uric acid with allopurinol slowed the progression of renal disease in patients with hyperuricemia and CKD [25]. Similarly, Kanbay et al. found improvements in endothelial function and renal parameters following uric acid reduction [26]. CKD-FIX trial investigators found that in patients with chronic kidney disease and a high risk of progression, urate-lowering treatment with allopurinol did not slow the decline in eGFR as compared with placebo [27]. In a study of 267 patients with type 1 diabetes and early-to-moderate diabetic kidney disease, allopurinol did not also have a beneficial effect in eGFR reduction [28]. Lastly, the FEATHER trial compared febuxostat versus placebo in CKD stage 3 patients, and again urate lowering did not mitigate kidney function decline [29].

Given the cross-sectional design of our study, causality cannot be inferred. Since the study did not track changes over time, it cannot establish a cause and effect relationship. As such, no firm conclusion can be drawn whether high SUA level is one of the causes of renal dysfunction and only a simple association between them is proved. It is possible that reduced eGFR may lead to decreased renal excretion of uric acid, resulting in secondary hyperuricemia. It is not uncommon to observe patients with CKD exhibiting higher SUA levels as CKD progresses. However, the consistency of experimental, epidemiologic, and interventional evidence supports a bidirectional relationship, where elevated SUA may both reflect and contribute to renal dysfunction [30]. Future longitudinal studies and randomized controlled trials are needed to clarify this relationship and assess whether early treatment of hyperuricemia in hypertensive patients can improve renal outcomes.

Our study has several strengths, including a relatively large sample size, a homogeneous population of untreated hypertensives and standardized measurements of biochemical and clinical parameters. However, it also has limitations. The cross-sectional nature precludes causal inferences as analyzed in the previous paragraph. Furthermore, residual confounding cannot be excluded since regression analysis with multivariate adjustment for potential confounders (age, sex, BMI, waist circumference, insulin resistance, lipid parameters) was not feasible. We did not assess dietary factors, purine intake or urinary uric acid excretion, all of which could influence SUA levels. Finally, we did not measure inflammatory markers or endothelial function, which could provide further mechanistic insights.

## Conclusions

In summary, our findings indicate that elevated serum uric acid levels may be associated with reduced renal function in untreated patients with arterial hypertension, although the correlation strength between SUA levels and eGFR was relatively weak. It is suggested that hyperuricemia may be a potentially modifiable risk factor in patients with hypertension and renal disease, but this cannot be postulated with certainty short of prospective cohort or interventional studies. Identifying and managing elevated SUA in hypertensive individuals could represent a novel strategy for mitigating long-term cardiovascular and renal risk. As said before, further research studies are warranted to explore whether uric acid-lowering therapy can improve outcomes in this high-risk population.
